# New Medical College

**Published:** 1860-07

**Authors:** 


A NEW MEDICAL COLLEGE, composed of ten professorships, is announced at Leavenworth City, Kansas Territory.

